# Addressing cognitive impairment in drivers on hemodialysis through reaction time

**DOI:** 10.1590/1980-5764-DN-2025-0382

**Published:** 2026-04-20

**Authors:** Áquilla dos Anjos Couto, Karla Carlos, Juliana Alencar Fuzinohara, Rubens Baptista, Matheus de Lima Gomes, Eduardo Nascimento de Lima, Gilmar Fernandes do Prado

**Affiliations:** 1 Universidade Federal de São Paulo, Escola Paulista de Medicina, Departamento de Neurologia e Neurocirurgia, São Paulo SP, Brazil.; 2 Universidade de São Paulo, Faculdade de Medicina, Hospital das Clínicas, São Paulo SP, Brazil.; 3 LIMHUB Serviços Tecnológicos Ltda, João Pessoa, PB, Brazil.

**Keywords:** Restless Legs Syndrome, Reaction Time, Cognition, Sleep Apnea, Obstructive, Public Health, Renal Insufficiency, Chronic, Síndrome das Pernas Inquietas, Tempo de Reação, Cognição, Apneia Obstrutiva do Sono, Saúde Pública, Insuficiência Renal Crônica

## Abstract

End-stage kidney disease affects cognition and is associated with sleep disorders (SD), both increasing reaction time (RT) and impairing decision-making capacity.

**Objective:**

To evaluate sleep disorders, age, dialysis duration, and education level on the RT of Brazilian drivers undergoing hemodialysis (HD) and the feasibility of the method employed.

**Methods:**

We used tablet-based software to test the RT of 35 drivers undergoing HD and 32 controls. We did a clinical interview, examination, and chart review. The data were summarized and analyzed, which included conducting multiple linear regression. Significance was considered for p<0.05.

**Results:**

RT evaluation was well-received and effortless to carry out. Most patients were male (82.9%); the mean age was 52 years; two-thirds had been on HD for less than four years; 47.5% had 9.5 years or less of education; 14.3% had restless legs syndrome/Willis-Ekbom disease (RLS/WED); 68.6% showed a high likelihood of obstructive sleep apnea (OSA). HD patients showed higher RT than controls (p<0.001), and RT was positively associated with age (p<0.001). Less than 9.5 years of education and being on dialysis for over four years are associated with longer RT (p<0.05). Those with RLS/WED had shorter RT (p<0.001), age remained a significant predictor in the regression model, and patients on HD had longer RT than controls (p<0.001).

**Conclusion:**

Testing the RT of drivers on HD in the clinic environment is fully feasible. Patients on HD had longer RT than controls. RLS/WED patients showed shorter RT, but, as age increases, the RT is longer.

## RESUMO

A doença renal terminal compromete a cognição e está associada a distúrbios do sono (DS), ambos elevando o tempo de reação (TR) e prejudicando a tomada de decisão.

**Objetivo:**

Avaliar o impacto da doença renal terminal, dos DS, idade, tempo de diálise e nível educacional no TR de motoristas brasileiros em hemodiálise (HD), além da viabilidade do método utilizado.

**Métodos:**

Utilizamos um aplicativo executado em *tablet* para testar o TR de 35 motoristas em HD e 32 controles. Realizamos entrevista clínica, exame físico e revisão de prontuários. Os dados foram analisados por regressão linear múltipla, considerando-se significante p<0,05.

**Resultados:**

A avaliação do TR foi bem aceita e de fácil aplicação. A maioria dos pacientes era do sexo masculino (82,9%); idade média de 52 anos; dois terços em HD há menos de quatro anos; 47,5% com escolaridade ≤9,5 anos; 14,3% apresentavam síndrome das pernas inquietas/doença de Willis-Ekbom (SPI/DWE); 68,6% com alta probabilidade de apneia obstrutiva do sono (AOS). Pacientes em HD apresentaram TR mais elevado que os controles (p<0,001), com associação direta entre TR e idade (p<0,001). Escolaridade ≤9,5 anos e HD >4 anos associaram-se a maior TR (p<0,05). Pacientes com SPI/DWE apresentaram TR menor (p<0,001). A idade manteve-se como preditor significativo no modelo.

**Conclusão:**

A avaliação do TR em motoristas em HD no ambiente clínico é viável. O TR é maior nesses pacientes e aumenta com a idade. SPI/DWE associou-se a TR reduzido.

## INTRODUCTION

Chronic kidney disease (CKD) affects >800 million patients worldwide^1^, impairing many body functions, mostly the nervous system where the buildup of toxins can lead to conditions such as altered levels of consciousness, seizures, paresthesia, peripheral neuropathies, and cognitive disorders^2^, which can limit or impair the ability to drive^3^.

Sleep disorders, such as insomnia, obstructive sleep apnea (OSA), and restless legs syndrome (RLS), are common in people with chronic kidney disease (CKD) and can worsen neuropsychological functions^4,5^.

Each hemodialysis session lasts 4 hours, and patients attend the treatment center three times a week, totaling 12 hours weekly, excluding travel^6^. Consequently, many patients drive themselves to the center, as Brazilian laws permit it with a valid driver's license and medical oversight.

Being a good driver requires more than just technical skills. It involves attention, quick decision-making, anticipating traffic scenarios, driving defensively, and respecting traffic laws. Conditions like CKD and sleep disorders can impair these essential driving skills^7^.

Sleep disorders affect 45 to 80% of patients with CKD, leading to issues like circadian rhythm changes, insomnia, fragmented sleep, RLS, and excessive daytime sleepiness^8^. These disorders can impair cognitive functions, including attention, emotional regulation, and memory, while increasing the risk of traffic accidents^9^, which prompts the need for a formal evaluation of capacity^10^.

Assessing a driver's reaction time to changing conditions is crucial. Various tests are designed for this, each with its own pros and cons. These tests measure the time taken to perceive a situation, process information, and respond to stimuli like light or sound^11^.

This study assessed the reaction time (RT) of hemodialysis (HD) patients in a dialysis clinic setting. The test measures participants’ psychomotor performance in milliseconds. We hypothesize that patients with CKD on HD will have a longer RT than controls, and those with CKD and sleep disorders will have a longer RT than patients with CKD without sleep disorders.

## METHODS

**Figure 1:**
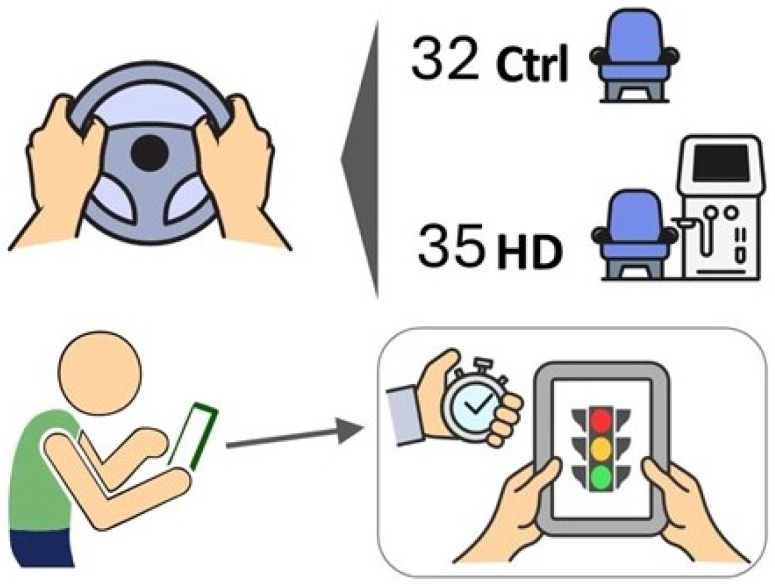
Sixty-seven drivers (32 control and 35 on hemodialysis) performed a reaction time test following a familiarization and learning session. All participants were instructed to touch the tablet's screen as soon as the green light appeared.

We examined the response time of drivers with CKD undergoing hemodialysis to react to a visual stimulus and complete a simple motor task, like tapping a tablet screen (simple psychomotor test, Figure 1). We aimed to interpret our results based on factors such as age, the existence of sleep disorders, duration of dialysis, level of education, and clinical and demographic variables.

### Study population and setting

The study involved 35 adult patients on renal replacement therapy via HD and 32 age-matched controls with normal renal function. All eligible hemodialysis patients who were invited to participate accepted enrollment, and no refusals were recorded. Participants aged 26 to 82 were included, while those with diabetic retinopathy, motor deficits, or who were in the first dialysis shift starting at 6:00 am were excluded to avoid potential sleep restriction effects. We collect demographic and clinical information such as gender, age, BMI, dialysis duration, education level, and current medications through interviews or medical records.

The patients underwent a traditional HD program lasting 4 hours per session, three times a week, between January and April 2024. The study was approved by the Research Ethics Committee of the Federal University of São Paulo — UNIFESP (# 5.282.165/2022), and all participants signed the consent form. The data is stored in a spreadsheet on the Research Electronic Data Capture (REDCap) platform, version 9.1.0.

### Reaction time

To measure RT and link the test to driving scenarios, we utilized a virtual traffic light with the standard red-yellow-green colors^12^ displayed on a tablet (Galaxy S6 Lite, Android 13, Samsung. Figure 1). Each participant received instructions from one of the researchers (AAC) on completing the test, and had a practice session to address any queries before the test. When the traffic light turns green, the participant touches the screen as quickly as possible. The time from the green light to the touch is the RT. The program records five attempts and calculates the raw data and average RT in milliseconds. We used the best RT and the average of the five attempts for our calculations.

### Sleep diary

We utilized a sleep diary to gather more specific data related to sleep. In this diary, participants were asked to report how they slept the night before the current HD day and the previous night if they could recall. We collected information on the total sleep time (TST), bedtime, wake-up time, sleep latency (SL), nocturnal awakenings, and the time awake after falling asleep (WASO).

### Sleepiness

We used the Epworth Sleepiness Scale (ESS) to evaluate daytime sleepiness and the Stanford Sleepiness Scale (SSS) to assess current sleepiness. A score of 10 or more on the ESS indicated the presence of sleepiness, while a score of 4 or more on the SSS suggested the participant felt somewhat foggy, which could be unsafe for activities like driving.

### Obstructive sleep apnea

Patients were evaluated for signs and symptoms commonly linked with OSA. We did not perform polysomnography. The evaluation considered high body mass index (BMI), a high-arched (ogival) palate, intense or harsh snoring, choking, and a Mallampati score of III or IV. Those with three or more of these characteristics suggestive of OSA were classified as having a high chance of OSA (HOSA), while those with fewer than three had a low chance of OSA (LOSA).

### Restless legs syndrome/Willis-Ekbom disease

We assessed patients for symptoms of RLS/WED according to the five criteria of the International Restless Legs Syndrome Study Group (IRLSSG)^13^. Patients were interviewed during the dialysis period, and those diagnosed with RLS/WED had their symptoms graded using the Restless Legs Syndrome Severity Scale (RLSSS), also from the IRLSSG, validated for Brazilian Portuguese^14^.

### Statistical analysis

The initial statistical analysis included summary measures. The inferential analyses involved Spearman's correlation, Mann-Whitney, Kruskal-Wallis, Dunn's multiple comparisons, and multiple linear regression. A significance level of 5% was used for all conclusions. Data were entered into Excel, and analyses were performed using Statistical Package for the Social Sciences — IBM-SPSS Statistics, version 24.

## RESULTS

The study involved 35 volunteers with CKD who were undergoing renal replacement therapy through HD. The average age of the participants was 52.2±14.1 years, and the majority were male (82.9%). The control group, which consisted of 32 individuals, had an average age of 53.1±12.5 years, which was not statistically different from the HD participants (p=0.86). Only 14.4% of the HD participants had a BMI above 30 kg/m^2^, and 45.7% had up to 9.5 years of education (Table 1). Hypertension was the main cause of CKD in 21 patients (60.0%); 11 participants (31.4%) had been on dialysis for more than four years. Additionally, 12 (34.3%) were in the 2^nd^ shift, and 23 (65.7%) were in the 3^rd^ shift (refer to Table 2).

**Table 1:** Main demographic data, sleep characteristics, and reaction time (seconds) of end-stage chronic kidney disease participants on hemodialysis.

Participant profile		n	M	SD	Md	Min	Max
Age (years)		35	52.2	14.1	52.4	26.5	82.5
Education (years)			9.4	3.6	11.0	4.0	16.0
BMI (Kg/m^2^)			25.2	4.9	24.0	18.8	40.8
Dialysis length (years)			4.0	3.6	2.8	0.8	15.3
Sleep diary							
	SL (min)	35	36.9	53.0	0.0	0.0	180.0
	TST (min)		410.6	137.4	420.0	210.0	720.0
	WASO (min)		15.4	36.6	0.0	0.0	150.0
	Score (0 to 10)		7.8	1.4	8.0	5.0	10.0
ESE			7.9	4.4	7.0	1.0	18.0
ESS			2.0	1.1	2.0	1.0	6.0
Reaction time (seconds)							
	Best performance	35	0.94	0.37	0.78	0.60	2.17
	Mean attempts		1.31	0.71	1.01	0.64	3.61
		**n**	**%**				
Sex							
	Female	6	17.1				
	Male	29	82.9				
Age range (years)							
	26 to 29	3	8.6				
	30 to 39	5	14.3				
	40 to 49	6	17.1				
	50 to 59	12	34.3				
	60 to 69	5	14.3				
	70 to 79	3	8.6				
	80 or more	1	2.8				
Education range (years)							
	1 to 5	7	20.0				
	6 to 10	10	28.6				
	≥11	18	51.4				
Education (years)							
	Up to 9.5	16	45.7				
	>9.5	19	54.3				
BMI ((Kg/m^2^)							
	18.5 to 24.9	20	57.1				
	25 to 29.9	10	28.6				
	30 to 34.9	3	8.6				
	35 to 39.9	1	2.9				
	≥40	1	2.9				

Abbreviations: N, Number; Min, Minimum; Max, Maximum; SD, Standard Deviation; BMI, Body Mass Index; HD, Hemodialysis; SL, Sleep Latency (minutes); TST, Total Sleep Time (minutes); WASO, Awake After Sleep Onset (minutes); Sleep Score, satisfaction with sleep (0, worst; 10, best); ESS, Epworth Sleepiness Scale; SSS, Stanford Sleepiness Scale.

**Table 2:** Probable etiology of end-stage kidney disease, regular medications used, degree of sleepiness, and high chance of obstructive sleep apnea.

Variable		Yes		No		Total	
		n	%	n	%	n	%
Etiology							
	Hypertension	21	60.0	14	40.0	35	100.0
	Diabetes mellitus	6	17.1	29	82.9		
	Nephritis	2	5.7	33	94.3		
	Polycystic kidney disease	4	11.4	31	88.6		
	Toxic	3	8.6	32	91.4		
	Recurrent pyelonephritis	2	5.7	33	94.3		
HD shift							
	2^nd^	12	34.3			35	100.0
	3^rd^	23	65.7				
Dialisys length duration (years)							
	1–2	17	48.6			35	100.0
	3–4	7	20.0				
	5–6	6	17.1				
	7–10	1	2.9				
	>10	4	11.4				
Medications							
	Antihypertensives	28	80.0	7	20.0	35	100.0
	Antidiabetics	5	14.3	30	85.7		
	NSAIDs	15	42.9	20	57.1		
	Corticosteroids	9	25.7	26	74.3		
	Antidepressants	12	34.3	23	65.7		
	Anxiolytics	7	20.0	28	80.0		
	Statins	3	8.6	32	91.4		
	Antiulcer medications	9	25.7	26	74.3		
	Antiasthmatics	18	51.4	17	48.6		
Sleep diary							
	Total sleep time (>7h)	17	48.6	18	51.4	35	100.0
	ESS≥10	11	31.4	24	68.6	35	100.0
	SSS>3	2	5.7	33	94.3	35	100.0
	RLS/WED	5	14.3	30	85.7	35	100.0
	HOSA	24	68.6	11	31.4	35	100.0

Abbreviations: DM, Diabetes Mellitus; HD, Hemodialysis; NSAIDs, Nonsteroidal Anti-Inflammatory Drugs; ESS, Epworth Sleepiness Scale; SSS, Stanford Sleepiness Scale; RLS/WED, Restless Legs Syndrome/Willis-Ekbom Disease; HOSA, high chance of OSA.

### Clinical profile associated with sleep

Eighteen (51.4%) had a TST ≥7 hours, 11 (31.4%) scored ≥10 on the ESS, and only 2 (5.7%) scored 4 or higher on the SSS.

Twenty-four individuals (68.6%) showed clinical signs of sleep-disordered breathing, including continuous and harsh snoring, choking, BMI >30 kg/m^2^, high-arched (ogival) palate, Mallampati III or IV, suggesting a HOSA.

RLS/WED was present in five (14.3%) patients (Table 2). Only one patient had a mild degree of RLS/WED, three had moderate, and one had severe symptoms.

### Chronic kidney disease versus controls

**Figure 2:**
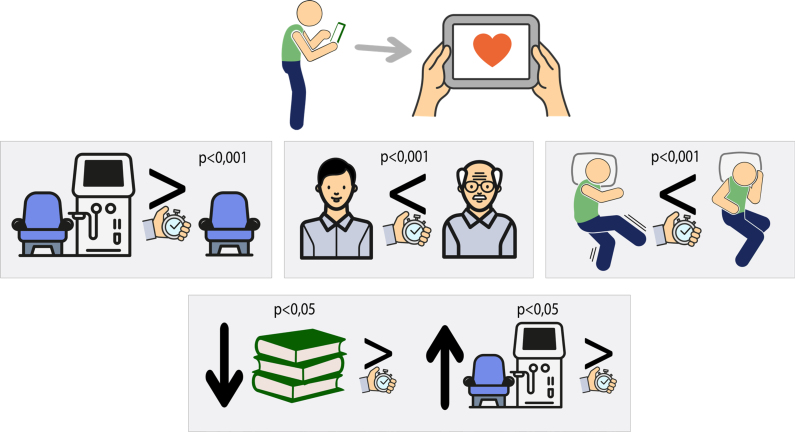
The reaction time test was well accepted by drivers on hemodialysis. However, they had significantly longer reaction time than controls. Older age, fewer years of education, and more years on hemodialysis were associated with longer reaction time, while restless legs syndrome/Willis-Ekbom disease was linked to shorter reaction time.

The best RT (best response) and the average RT of HD patients were higher than those of the control group. The average of the best RTs for the 35 individuals on HD was 0.94±0.37 seconds, while that of the control group was 0.71±0.06 seconds (p<0.001; Table 3, Figure 2). The average RT of the HD individuals was also higher than that of the control group (1.31±0.64 seconds; 0.84±0.14 seconds; p<0.001, respectively; Table 3, Figure 2).

**Table 3:** Values of reaction time (milliseconds) for the best performance and average attempts of participants with end-stage kidney disease on hemodialysis, by sex, dialysis length duration, educational level, and sleep parameters.

		n	Average	SD	Min	Max	Median	p-value
Best performance								
Sex								
	Female	6	0.73	0.12	0.62	0.92	0.72	0.120*
	Male	29	0.98	0.40	0.60	2.17	0.85	
Dialysis length duration (years)								
	≤4	23	0.84	0.26	0.60	1.51	0.71	0.049*
	>4	12	1.12	0.49	0.62	2.17	0.98	
Educational level ranges (years)								
	1 to 5	7	1.05	0.33	0.71	1.52	1.00	0.159†
	6 to 10	10	1.05	0.50	0.60	2.17	0.95	
	≥11	18	0.83	0.29	0.62	1.85	0.72	
Educational level								
	≤9.5	16	1.06	0.44	0.60	2.17	0.96	0.140*
	>9.5	19	0.84	0.29	0.62	1.85	0.72	
TTS (hours)								
	≤7	18	0.97	0.41	0.60	2.17	0.85	0.830*
	>7	17	0.90	0.34	0.63	1.85	0.77	
ESSE								
	<10	24	0.97	0.42	0.60	2.17	0.83	0.569*
	≥10	11	0.86	0.25	0.62	1.30	0.72	
ESS								
	≤3	33	0.93	0.38	0.60	2.17	0.77	0.227*
	>3	2	1.11	0.27	0.92	1.30	1.11	
HOSA								
	No	1	0.96	0.43	0.64	1.85	0.70	0.545*
	Yes	24	0.93	0.35	0.60	2.17	0.83	
RLS/WED								
	No	30	0.99	0.38	0.62	2.17	0.86	0.001*
	Yes	5	0.64	0.03	0.60	0.69	0.63	
Insomnia								
	No	22	0.91	0.38	0.60	2.17	0.75	0.473*
	Yes	13	0.98	0.37	0.62	1.85	0.89	
Average attempts								
Sex								
	Female	6	0.99	0.23	0.74	1.34	0.92	0.335*
	Male	29	1.38	0.76	0.64	3.61	1.05	
Time-HD								
	≤4	23	1.16	0.56	0.64	2.80	0.90	0.054*
	>4	12	1.61	0.89	0.78	3.61	1.18	
Educational level ranges (years)								
	1 to 5	7	1.73	0.64	0.88	2.69	1.75	0.032†
	6 to 10	10	1.50	0.96	0.64	3.61	1.21	
	≥11	18	1.05	0.46	0.66	2.44	0.89	
Educational level								
	≤9.5	16	1.63	0.85	0.64	3.61	1.40	0.020*
	>9.5	19	1.05	0.45	0.66	2.44	0.90	
TST (hours)								
	≤7	18	1.26	0.77	0.64	3.61	0.99	0.409*
	>7	17	1.38	0.66	0.74	2.80	1.05	
ESS								
	<10	24	1.33	0.73	0.64	3.61	1.03	0.749*
	≥10	11	1.29	0.71	0.74	2.80	0.98	
SSS								
	≤3	33	1.27	0.68	0.64	3.61	0.98	0.177*
	>3	2	2.00	1.14	1.19	2.80	2.00	
HOSA								
	No	11	1.13	0.57	0.74	2.44	0.88	0.183*
	Yes	24	1.40	0.77	0.64	3.61	1.15	
RLS/WED								
	No	30	1.40	0.73	0.74	3.61	1.08	0.014*
	Yes	5	0.83	0.29	0.64	1.34	0.74	
Insomnia								
	No	22	1.29	0.74	0.64	3.61	0.98	0.746*
	Yes	13	1.36	0.69	0.66	2.80	1.05	

Abbreviations, ESKD, End-Stage Kidney Disease; HD, Hemodialysis; RT, Reaction Time; TST, Total Sleep Time; ESS, Epworth Sleepiness Scale; SSS, Stanford Sleepiness Scale; HOSA, high chance of OSA; RLS/WED, Restless Legs Syndrome/Willis-Ekbom Disease; Min, Minimum; Max, Maximum, SD, Standard Deviation.

* Mann-Whitney;

† Kruskal-Wallis.

### Reaction time and sleep disorders

Patients with HOSA or LOSA did not differ statistically in terms of RT. The average of the best RTs and the average RTs of HD patients with HOSA compared to those with LOSA were 0.93±0.35 seconds, p=0.545; 1.40±0.77 seconds, p=0.183, respectively.

The RT of participants with RLS/WED (best RT and average RT) was shorter than that of participants without RLS/WED (p<0.001; Table 3, Figure 2). We did not observe a statistically significant association between RT in patients with or without insomnia, EDS, ESS, ESS, and TST.

### Dialysis treatment length

HD patients tended to show a progressive increase in RT the longer they were on HD. Over time, RT increases, and, after four years of HD, the difference in performance is statistically significant (p=0.049). The average attempts also showed a trend toward poorer results after four years of HD (p=0.054; Table 3, Figure 2).

### Education level

Regarding the education level of patients and the average number of attempts at RT, patients with more than 9.5 years of education showed better results than patients with less than 9.5 years of education (p=0.02). The best RT did not exhibit a statistically significant difference in relation to education level (p=0.159; Table 3, Figure 2).

### Age

Both the best RT and the average RT varied inversely with age; the older the age, the higher the RT value (p<0.001), meaning that older HD individuals responded more slowly (Table 3, Figure 2).

### Multivariate analysis of reaction time

To evaluate the combined impact of several variables of interest in this research on RT (best performance and average attempts), we performed an analysis through multiple linear regression model adjustments. Only variables that demonstrated statistical significance, with p-values of up to 0.100 (10%) in the univariate analyses in relation to reaction time, were included in this adjustment process.

The various adjustments revealed that the patient's age remains relevant in the presence of education level, dialysis treatment length, and the presence of RLS/WED to explain the best performance in the reaction test (models A and B; Table 4). Similarly, this pattern repeats for the average attempts, where education level, dialysis treatment length, and the presence of RLS/WED lose their influence in favor of age (models C, D, and E; Table 4).

**Table 4:** Reaction time (milliseconds) in the group of 35 participants with end-stage kidney disease on hemodialysis, and in the subgroups low chance of obstructive sleep apnea, high chance of obstructive sleep apnea, and control group.

Group		Age	Best RT	RT average
ESKD with LOSA (n=11)				
	Average	49.6	0.96	1.13
	Standard deviation	14.8	0.43	0.57
	Median	49.0	0.70	0.88
	Minimum	26.0	0.64	0.74
	Maximum	78.0	1.85	2.44
ESKD with HOSA (n=24)				
	Average	53.2	0.93	1.40
	Standard deviation	13.9	0.35	0.77
	Median	54.5	0.83	1.15
	Minimum	29.0	0.60	0.64
	Maximum	82.0	2.17	3.61
	p-value	0.813*	0.032*	0.001*

Abbreviations, ESKD, end-stage kidney disease; HD, hemodialysis; RT, reaction time; LOSA, low chance of OSA; HOSA, high chance of OSA.

* Kruskal-Wallis.

## DISCUSSION

The primary objective of this study was to evaluate RT using a low-cost, easily accessible instrument suitable for use in dialysis clinics. Additionally, the study aimed to examine the impact of sleep disorders on RT among drivers with CKD who are undergoing hemodialysis. While various methods for measuring RT exist, many are expensive, technically complex, and not well suited to the practical constraints of healthcare settings. Therefore, a simple and accessible technology that can be utilized by nurses, physicians, and other healthcare professionals working with hemodialysis patients would be welcome. Such screening tools could enhance clinical decision-making and support public health interventions aimed at mitigating risks for this vulnerable population and others they may affect while driving.

### Clinical and demographic data

Most patients have been on dialysis for less than four years, and the most common etiologies of CKD were hypertension and diabetes mellitus (DM). Although these are independently associated with severe brain diseases, including dementia^15^, we did not observe a statistically significant difference in RT between patients with or without these conditions in our sample. This could be due to the small sample size, which may not be sufficient to detect a slight difference.

In our study, we included patients who undergo dialysis during the second and third shifts and deliberately excluded those in the first. This decision aimed to minimize the confounding effect of acute sleep restriction, which is common in first-shift patients due to very early treatment start times and is independently associated with impaired attention and prolonged reaction time^16^. Although first-shift patients may represent a subgroup with a higher burden of sleep disorders, their inclusion could have introduced an additional bias related to acute sleep deprivation and its potential interaction with uremic-related cognitive dysfunction, making it difficult to disentangle chronic and acute effects on RT. This combined effect represents a relevant research question that deserves investigation in a dedicated study.

### Sleep

Sleep disorders were prevalent, as is characteristic of this population. Insomnia occurred in more than a third of the patients, decreased TST in more than half, EDS in a third, at least two-fifths snored, and 14.3% had RLS/WED, as observed in Table 1.

Considering the relationship between OSA and traffic accidents, a sleep specialist clinically identified patients with a high chance of OSA in the CKD group, noting that most presented characteristics indicative of OSA, such as Mallampati III or IV, snoring, choking, and some had a BMI above 30 kg/m². Furthermore, CKD itself is epidemiologically associated with OSA, with a prevalence of 57% in non-dialytic patients and 49% in dialytic patients^17^. Although polysomnography is the most reliable method for diagnosing OSA, we did not perform it on these patients. However, a complete clinical evaluation is an excellent predictor of OSA, with high sensitivity and specificity compared to the STOP-Bang questionnaire^18^.

Patients who manifested sleepiness did not have a higher RT. Still, there is data showing that RT in OSA patients increases with hypoxic load or micro-awakenings. The presence of sleepiness is not relevant when age is considered in the analysis model^19^.

While our study and some others did not find a significant link between poor sleep quality and slower RT, most studies indicate that better sleep quality is linked to faster RT and improved task performance^18^. The varying perceptions of sleep quality can be complicated by different types of insomnia (such as normal or short sleep duration), leading to biases in how patients are grouped for analysis. It is possible that this might have affected our sample, as we did not use more precise methods to diagnose these patients^20,21^.

### Chronic kidney disease and controls

Any instrument measuring RT is subject to variations intrinsic to the equipment itself. Therefore, it is not advisable to compare data obtained through different methods, which requires researchers to strictly use the same equipment and methods generated by another researcher^22^. We applied the same procedure to a group of age-matched volunteers with normal renal function, who presented a significantly lower RT, with a statistically significant difference. This finding shows that the method used is capable of discriminating between two clinically different groups.

### Restless legs syndrome/Willis-Ekbom disease

In univariate analysis, patients with CKD and RLS/WED had a shorter RT than those without RLS/WED. Initially, we understood that this difference could be explained by a previously identified higher brain processing speed in patients with RLS/WED^23^. However, this difference disappeared in the multivariate analysis when age was introduced into the model (Table 5). Since secondary RLS often occurs in older patients^24^, it is possible that this concomitance attenuated the strength of the association with RLS/WED in a small sample, with only five patients having RLS/WED. This finding deserves further careful evaluation in future studies.

**Table 5:** Adjustments of Multiple Linear Regression Models to Estimate Average Attempts/Best Performance Based on Age, Restless Legs Syndrome/Willis-Ekbom Disease, Educational Level, and Duration of Dialysis.

			Coefficient	Standard error (coefficient)	t-statistic	p-value
**Best RT (milliseconds)**	**model A**	Age (years)	0.016	0.005	3.649	0.001
		RLS/WED	-0.040	0.165	-0.240	0.812
		Educational level (years)	0.000	0.015	-0.016	0.987
		Dialysis length duration (≤ 2 years > 2 years)	0.106	0.105	1.014	0.319
		(Constant)	-0.087	0.369	-0.236	0.815
	**model B**		**Coefficient**	**Standard error (coefficient)**	**t-statistic**	**p-value**
		Age (years)	0.015	0.005	3.206	0.003
		RLS/WED	-0.045	0.164	-0.278	0.783
		Educational level (years)	-0.004	0.016	-0.286	0.777
		Dialysis length duration (≤ 4 years > 4 years)	0.133	0.111	1.194	0.242
		(Constant)	0.016	0.339	0.047	0.963
RT Average (milliseconds)	**model C**		**Coefficient**	**Standard error (coefficient)**	**t-statistic**	**p-value**
		Age (years)	0.037	0.008	4.728	<0.001
		RLS/WED	0.137	0.269	0.510	0.614
		Dialysis length duration (≤ 4 years > 4 years)	0.114	0.183	0.626	0.536
		Educational level (years)	-0.023	0.025	-0.899	0.376
		(Constant)	-0.559	0.556	-1.004	0.323
	**model D**		**Coefficient**	**Standard error (coefficient)**	**t-statistic**	**p-value**
		Age (years)	0.038	0.008	4.789	<0.001
		RLS/WED	0.159	0.269	0.593	0.558
		Dialysis length duration (≤ 4 years > 4 years)	0.098	0.182	0.538	0.594
		Educational level (1–5, 6–10 ≥11)	-0.073	0.117	-0.622	0.539
		(Constant)	-0.635	0.596	-1.064	0.296
	**model E**		**Coefficient**	**Standard error (coefficient)**	**t-statistic**	**p-value**
		Age (years)	0.035	0.008	4.517	<0.001
		RLS/WED	0.058	0.272	0.214	0.832
		Dialysis length duration (≤ 4 years > 4 years)	0.141	0.180	0.785	0.439
		Educational level (≤ 9.5 years >9.5years)	-0.268	0.181	-1.482	0.149
		(Constant)	-0.274	0.578	-0.475	0.638

Abbreviations, RLS/WED, Restless Legs Syndrome/Willis-Ekbom Disease; HD, Hemodialysis; RT, Reaction Time.

### Dialysis length time and education level

Patients who had been on dialysis for more than four years had a higher RT than those with less time on dialysis. Similarly, individuals with lower education levels had a higher RT. However, these differences disappeared when age was taken into account in the multivariate regression model.

It is known that CKD is an independent factor for dementia and mortality^25,26^. Therefore, other study models and more representative samples are needed to clarify whether a simple and practical test like RT, which can be easily incorporated into medical records for longitudinal evaluation of each patient, can serve as an indicator of more significant brain impairment or as a reference for preventive interventions in the brain's degenerative process in this population.

Higher education is associated with greater cognitive reserve^27^, which could justify a lower RT (better performance) in those with more education. However, this prediction has not been consistently demonstrated in HD patients, suggesting that these patients may be subject to different pathophysiological phenomena resulting from the severe impairment of the kidney-brain axis^5,28^.

### Age

Our data indicates that RT increases with age. However, the link between aging and longer RT in drivers remains contentious, with variations depending on the testing environment (simulated or real) and even the specific country where the study was carried out. When age is considered outside of a specific context, as in our study, RT consistently increases with age, justifying our data where age remained an explanatory variable in all regression models, with other significant variables in univariate analysis.

In more realistic scenarios of driver RT assessment, there is no clear relationship between increasing age and higher RT in experiments involving surprises, such as an object being thrown onto the road or a person suddenly crossing it^11^. In anticipated scenarios, the RT is shorter and younger drivers have better performance^11^. Knowing something is about to happen activates alert mechanisms, perception, and response processing, keeping the individual ready and prepared to brake or swerve. However, more experienced drivers, in real tests, apparently perceive dangerous situations more easily, taking into account, for example, environmental circumstances that make prediction difficult. This automatically induces them to anticipate physical and mental surprises, responding more quickly, as if experiencing an expected situation, reducing their tendency to have a higher RT^11,29^.

### Weaknesses

Our sample size is small, and we did not conduct polysomnography, which would have allowed for a more accurate diagnosis of OSA. Due to the limited sample size, we were also unable to assess the independent impact of diabetes and hypertension on RT, although both conditions are themselves associated with cognitive impairment^30,31^.

The small sample may have limited the statistical power to detect more subtle cognitive effects, including potential ceiling effects in attention-based measures such as RT. Although sleep disorders are known to impair attention and cognitive performance, this association was not consistently demonstrated in our sample clinically diagnosed with sleep disturbances, except in the small subgroup with RLS/WED, which showed shorter RTs.

Although the study relied on a convenience sample, all eligible patients who were invited to participate accepted enrollment, and no refusals were recorded. Therefore, the potential for selection bias related to self-exclusion of patients with more severe cognitive impairment or reluctance to drive may be lower than typically assumed. Nevertheless, the non-probabilistic nature of the sample limits generalizability, and larger, representative studies are needed to confirm these findings.

A larger sample would also allow a more robust evaluation of the effects of education level and duration of hemodialysis on RT, particularly given that these variables showed age dependency in the regression models^27,29,31^.

In conclusion, a tablet-based RT test is a viable tool in the cognitive assessment of drivers with CKD on HD. It increases with age and years undergoing dialysis and is lower in those with higher education levels and those diagnosed with RLS/WED. Age plays an important role in these associations and remains the only explanation for RT variation in the multivariate analysis, suggesting that significant associations in the univariate analysis need confirmation in studies with more participants. Using RT for drivers on HD is practical, cost-effective, and can serve as a valuable psychomotor assessment in dialysis clinics and at driver's license tests. It aligns with the digital revolution, allowing for continuous monitoring of brain health in HD patients, educating patients and families, and helping prevent and treat cognitive decline in RT patients.

## Data Availability

The datasets generated and/or analyzed during the current study are available from the corresponding author upon request.

## References

[1] Jha V, Garcia-Garcia G, Iseki K, Li Z, Naicker S, Plattner B (2013). Chronic kidney disease: global dimension and perspectives. Lancet.

[2] Drew DA, Weiner DE, Sarnak MJ (2019). Cognitive impairment in CKD: pathophysiology, management, and prevention. Am J Kidney Dis.

[3] Skroeder NR, Jacobson SH, Lins LE, Kjellstrand CM (1994). Acute symptoms during and between hemodialysis: the relative role of speed, duration, and biocompatibility of dialysis. Artif Organs.

[4] Oliveira MM, Conti CF, Valbuza JS, Carvalho LBC, Prado GF (2010). The pharmacological treatment for uremic restless legs syndrome: evidence-based review. Mov Disord.

[5] Viggiano D, Wagner CA, Martino G, Nedergaard M, Zoccali C, Unwin R (2020). Mechanisms of cognitive dysfunction in CKD. Nat Rev Nephrol.

[6] Ritt GF, Braga PS, Guimarães EL, Bacelar T, Schriefer A, Kraychete AC (2007). Terapia renal substitutiva em pacientes do interior da Bahia: avaliação da distância entre o município de moradia e a unidade de hemodiálise mais próxima. J Bras Nefrol.

[7] Owsley C (2004). Transportation in an aging society: a decade of experience.

[8] De Santo RM, Perna A, Di Iorio BR, Cirillo M (2010). Sleep disorders in kidney disease. Minerva Urol Nefrol.

[9] Dorrian J, Centofanti S, Smith A, McDermott KD (2019). Self-regulation and social behavior during sleep deprivation. Prog Brain Res.

[10] Charles L, Molnar F, Frank C (2025). When and how to assess decision-making capacity in your patients. Can Fam Physician.

[11] Green M (2000). "How long does it take to stop?" Methodological analysis of driver perception-brake times. Transportation Human Factors.

[12] Gomes ML, Lima EN, Carlos K, Prado GF (2024). Neuro-Sono 1.0 Traffic Light Reaction Time Testing.

[13] Allen RP, Picchietti DL, Garcia-Borreguero D, Ondo WG, Walters AS, Winkelman JW (2014). Restless legs syndrome/Willis-Ekbom disease diagnostic criteria: updated International Restless Legs Syndrome Study Group (IRLSSG) consensus criteria--history, rationale, description, and significance. Sleep Med.

[14] Masuko AH, Carvalho LBC, Machado MAC, Morais JF, Prado LBF, Prado GF (2008). Translation and validation into the Brazilian Portuguese of the restless legs syndrome rating scale of the International Restless Legs Syndrome Study Group. Arq Neuropsiquiatr.

[15] Mulligan MD, Murphy R, Reddin C, Judge C, Ferguson J, Alvarez-Iglesias A (2023). Population attributable fraction of hypertension for dementia: global, regional, and national estimates for 186 countries. EClinicalMedicine.

[16] Whittall H, Pillion M, Gradisar M (2018). Daytime sleepiness, driving performance, reaction time and inhibitory control during sleep restriction therapy for Chronic Insomnia Disorder. Sleep Med.

[17] Pires LA, Paula RB, Ezequiel DGA, Fernandes NMS Disfunção cognitive em doença renal crônica pré-dialítica: uma revisão sistemática. Revista Neurociências.

[18] Fonseca LBM, Silveira EA, Lima NM, Rabahi MF (2016). STOP-Bang questionnaire: translation to Portuguese and cross-cultural adaptation for use in Brazil. J Bras Pneumol.

[19] Pahari P, Korkalainen H, Karhu T, Arnardottir ES, Töyräs J, Leppänen T (2024). Reaction time in psychomotor vigilance task is related to hypoxic load in males with sleep apnea. J Sleep Res.

[20] Kapur VK, Auckley DH, Chowdhuri S, Kuhlmann DC, Mehra R, Ramar K (2017). Clinical practice guideline for diagnostic testing for adult obstructive sleep apnea: an american academy of sleep medicine clinical practice guideline. J Clin Sleep Med.

[21] Barreto LA, Coin-Carvalho JE, Carvalho LBC, Prado LBF, Prado GF (2014). Psychosocial features of Brazilian patients with paradoxical insomnia: a qualitative study. J Sleep Disor: Treat Care.

[22] Brenner E, Smeets JBJ How can you best measure reaction times? J Mot Behav. 2019.

[23] Magalhaes SC, Paiva JPQ, Kaelin-Lang A, Sterr A, Luiz Eckeli A, Winkler AM (2019). Short-interval intracortical inhibition is decreased in restless legs syndrome across a range of severity. Sleep Med.

[24] Allen RP, Earley CJ (2000). Defining the phenotype of the restless legs syndrome (RLS) using age-of-symptom-onset. Sleep Med.

[25] Murray AM, Knopman DS (2010). Cognitive impairment in CKD: no longer an occult burden. Am J Kidney Dis.

[26] Griva K, Stygall J, Hankins M, Davenport A, Harrison M, Newman SP (2010). Cognitive impairment and 7-year mortality in dialysis patients. Am J Kidney Dis.

[27] Kremen WS, Elman JA, Panizzon MS, Eglit GML, Sanderson-Cimino M, Williams MK (2022). Cognitive reserve and related constructs: a unified framework across cognitive and brain dimensions of aging. Front Aging Neurosci.

[28] van Zwieten A, Wong G, Ruospo M, Palmer SC, Teixeira-Pinto A, Barulli MR (2019). Associations of cognitive function and education level with all-cause mortality in adults on hemodialysis: findings from the COGNITIVE-HD study. Am J Kidney Dis.

[29] Lerner ND (1993). Brake perception-reaction times of older and younger drivers. Proc Hum Factors Ergon Society Annual Meeting.

[30] AlHejaili F, Hashmi MN, Alsuwaida A, Ankawi GA, Alsuwaida AA, AlZahrani MT (2024). Driving safety among patients with end-stage renal disease on hemodialysis. Fortune J Health Sci.

[31] Makishita H, Matsunaga K (2008). Differences of drivers’ reaction times according to age and mental workload. Accid Anal Prev.

